# Effects of rapamycin and curcumin on inflammation and oxidative stress in vitro and in vivo — in search of potential anti-epileptogenic strategies for temporal lobe epilepsy

**DOI:** 10.1186/s12974-018-1247-9

**Published:** 2018-07-23

**Authors:** C. M. Drion, J. van Scheppingen, A. Arena, K. W. Geijtenbeek, L. Kooijman, E. A. van Vliet, E. Aronica, J. A. Gorter

**Affiliations:** 1 Center for Neuroscience, Swammerdam Institute for Life Sciences, University of Amsterdam, Amsterdam, The Netherlands; 2 Department of (Neuro) Pathology, Amsterdam UMC, University of Amsterdam, Amsterdam, The Netherlands; 3 Department of Biochemical Sciences, Sapienza University of Rome, Rome, Italy; 4 Stichting Epilepsie Instellingen Nederland (SEIN), Heemstede, The Netherlands

**Keywords:** Rapamycin, Curcumin, Inflammation, Oxidative stress, Temporal lobe epilepsy, mTOR, MAPK

## Abstract

**Background:**

Previous studies in various rodent epilepsy models have suggested that mammalian target of rapamycin (mTOR) inhibition with rapamycin has anti-epileptogenic potential. Since treatment with rapamycin produces unwanted side effects, there is growing interest to study alternatives to rapamycin as anti-epileptogenic drugs. Therefore, we investigated curcumin, the main component of the natural spice turmeric. Curcumin is known to have anti-inflammatory and anti-oxidant effects and has been reported to inhibit the mTOR pathway. These properties make it a potential anti-epileptogenic compound and an alternative for rapamycin.

**Methods:**

To study the anti-epileptogenic potential of curcumin compared to rapamycin, we first studied the effects of both compounds on mTOR activation, inflammation, and oxidative stress in vitro, using cell cultures of human fetal astrocytes and the neuronal cell line SH-SY5Y. Next, we investigated the effects of rapamycin and intracerebrally applied curcumin on status epilepticus (SE)—induced inflammation and oxidative stress in hippocampal tissue, during early stages of epileptogenesis in the post-electrical SE rat model for temporal lobe epilepsy (TLE).

**Results:**

Rapamycin, but not curcumin, suppressed mTOR activation in cultured astrocytes. Instead, curcumin suppressed the mitogen-activated protein kinase (MAPK) pathway. Quantitative real-time PCR analysis revealed that curcumin, but not rapamycin, reduced the levels of inflammatory markers IL-6 and COX-2 in cultured astrocytes that were challenged with IL-1β. In SH-SY5Y cells, curcumin reduced reactive oxygen species (ROS) levels, suggesting anti-oxidant effects. In the post-SE rat model, however, treatment with rapamycin or curcumin did not suppress the expression of inflammatory and oxidative stress markers 1 week after SE.

**Conclusions:**

These results indicate anti-inflammatory and anti-oxidant properties of curcumin, but not rapamycin, in vitro. Intracerebrally applied curcumin modified the MAPK pathway in vivo at 1 week after SE but failed to produce anti-inflammatory or anti-oxidant effects. Future studies should be directed to increasing the bioavailability of curcumin (or related compounds) in the brain to assess its anti-epileptogenic potential in vivo.

**Electronic supplementary material:**

The online version of this article (10.1186/s12974-018-1247-9) contains supplementary material, which is available to authorized users.

## Background

Temporal lobe epilepsy (TLE) is the most common form of acquired epilepsy in adults [1]. TLE is characterized by progressive development of spontaneous seizures after an initial insult, often associated with hippocampal sclerosis, mossy fiber sprouting, and blood-brain barrier dysfunction [2]. About 30% of TLE patients do not respond to treatment with anti-epileptic drugs (AEDs) that are used to suppress seizures [3, 4]. Therefore, there is a need to develop treatments that interfere with epileptogenic mechanisms (anti-epileptogenic strategies).

In recent years, the mammalian target of rapamycin (mTOR) pathway has been studied as a possible target for anti-epileptogenic strategies [5–7]. The mTOR pathway regulates a large number of cellular processes [8, 9], and mTOR hyperactivation occurs in TLE patients and in several experimental models for epilepsy [10]. Several studies have shown potential anti-epileptogenic properties of mTOR pathway inhibitor rapamycin in different experimental models of TLE [11–14].

Still, the mechanisms mediating possible anti-epileptogenic effects of rapamycin remain to be elucidated. In the electrical post-status epilepticus (SE) rat model for TLE, where the SE is the equivalent of the initial insult, it was shown that chronic treatment with the mTOR inhibitor rapamycin suppressed spontaneous seizures and reduced cell death, mossy fiber sprouting, and blood-brain barrier leakage after SE [14]. Since rapamycin was able to reduce SE-induced microgliosis in rats after pilocarpine-induced SE [15] and kainic acid-induced SE [16] and has been shown to be neuroprotective after traumatic brain injury [17, 18] and stroke [19], it was proposed that rapamycin might have anti-epileptogenic effects through anti-inflammatory actions, possibly mediated by inhibition of the mTOR pathway.

However, rapamycin is effective in suppressing epileptogenesis only when treatment is continued, and rapamycin blood levels remain sufficiently high [20]. Moreover, prolonged treatment with rapamycin produces unwanted side effects, primarily on growth [14, 20, 21]. Therefore, there is a growing interest for alternative anti-epileptogenic treatments through mTOR inhibition. In this context, curcumin is considered. Curcumin is the main component of turmeric from the *Curcuma longa* plant. It is known for anti-inflammatory and neuroprotective properties [22–24], but it has also been reported to inhibit the mTOR pathway [25] and the mitogen-activated kinase (MAPK) pathways (extracellular signal-regulated kinase (ERK)1/2 and p38 pathway) [26]. In addition, curcumin has anti-oxidant effects [23, 27, 28]. No adverse effects of curcumin have been reported in phase 1 clinical studies [29, 30]. Because of its rapid degradation, curcumin has a low bioavailability in vivo [31] which could pose a challenge for its use as an anti-epileptogenic drug. Still, its anti-inflammatory, anti-oxidant, and mTOR-inhibiting properties make curcumin potentially anti-epileptogenic and possibly an interesting alternative to rapamycin.

Here, we aim to elucidate anti-inflammatory and anti-oxidant effects of curcumin compared to rapamycin in the context of epileptogenesis. We first studied the effects of both compounds on inflammation in vitro. Next, we studied anti-inflammatory and anti-oxidant effects of rapamycin and curcumin in vivo, in the early phase of epileptogenesis after SE in rats. With this combined approach, we aim to shed light on the anti-epileptogenic potential of curcumin compared to rapamycin and study the possible anti-inflammatory and anti-oxidant actions as potential underlying mechanisms.

## Methods

### Effects of rapamycin and curcumin on inflammation and oxidative stress in vitro

To assess the effects of rapamycin and curcumin on inflammation in vitro, we used primary human fetal astrocyte cell cultures and studied the levels of pro-inflammatory cytokines after challenging the cultures with interleukin 1-β (IL-1β). To study the effects of curcumin on oxidative stress in vitro, we studied the reactive oxygen species (ROS) levels in human primary neuronal cultures.

#### Astrocyte cell cultures

Primary astrocyte-enriched cell cultures were made from human fetal brain tissue (cortex, 14–19 gestational weeks) obtained from medically induced abortions. A written informed consent for the use of the tissue for research purposes was given by all donors to the Bloemenhove Clinic. The tissue was obtained in accordance with the Declaration of Helsinki and the Academic Medical Center (AMC) Research Code provided by the Medical Ethics Committee of the AMC. Cell isolation was performed as described in Additional file 1 and elsewhere [32]. Cultures were incubated with Dulbecco’s modified Eagle’s medium (DMEM)/HAM F10 (1:1) medium (Gibco, Life Technologies, Grand Island, NY, USA), supplemented with 1% penicillin/streptomycin and 10% fetal calf serum (FCS; Gibco, Life Technologies, Grand Island, NY, USA). Cultures were refreshed twice a week and reached confluence after 2–3 weeks. Secondary astrocyte cultures for experimental manipulation were established by trypsinizing confluent cultures and re-plating onto poly-l-lysine (PLL; 15 μg/ml, Sigma-Aldrich; St. Louis, MO, USA)-precoated 12 and 24-well plates (Costar, Cambridge, MA, USA; 10 × 10^4^ cells/well in a 12-well plate for RNA isolation and quantitative real-time PCR; 5 × 10^4^ cells/well in a 24-well plate for immunocytochemistry). Astrocytes were used for analyses at passages 2–4. Cell cultures were stimulated with human recombinant (r)IL-1β (Peprotech, Rocky Hill, NJ, USA; 10 ng/ml) for 24 h. Treatment of astrocytes with rapamycin (100 nM) or curcumin (10 μM) in 0.05% dimethyl sulfoxide (DMSO) was either started 24 h before, and continued during IL-1β stimulation (pre-treatment), or given simultaneously with IL-1β stimulation (simultaneous treatment). Concentrations of rapamycin (100 nm) and curcumin (10 μM) were selected based on previous work [33–36], and we selected the concentrations used in the experiments (100 nm rapamycin and 10 μM curcumin) after testing multiple concentrations with cell viability assays—see Additional file 1 and  Additional file 2: Figure S1). Cells were harvested 24 h after stimulation with IL-1β.

#### Human neuronal culture

To study the effects of curcumin on oxidative stress, we used the human neuroblastoma SH-SY5Y cell line, which is widely used for studying oxidative stress in vitro [37]. SH-SY5Y cells were cultured in Dulbecco’s modified Eagle’s medium (DMEM)/HAM F12 (1:1) (Gibco, Life Technologies, Grand Island, NY, USA) supplemented with 1% penicillin/streptomycin and 10% FCS (Gibco, Life Technologies, Grand Island, NY, USA). The cells were seeded into 96-well cell culture plates at a density of 10 × 10^3^ cells per well and allowed to adhere for 24 h in a 5% CO_2_ incubator at 37 °C. The culture medium was then replaced with either fresh medium containing 0.05% DMSO alone (vehicle) or with different concentrations of curcumin (1, 5, 10, and 20 μM) in 0.05% DMSO, and cell cultures were incubated in a 5% CO_2_ incubator for 30 min. For cell viability analysis, see Additional file 1 and  Additional file 3: Figure S2).

#### Oxidative stress assay

Intracellular ROS levels were measured in SH-SY5Y cells after treatment with the different concentrations (1, 5, 10, and 20 μM) of curcumin using the 2′7′-dichlorofluorescein (DCF, Sigma-Aldrich, St Louis, MO, USA) method, which is described in Additional file 1. The formation of DCF due to the ROS-driven oxidation of H2DCFH was measured using a microplate reader with excitation and emission wavelengths of 485 nm (bandwidth 5 nm) and 535 nm (bandwidth 5 nm), respectively. In this assay, the levels of DCF fluorescence are directly proportional to intracellular ROS levels and reported as a fold change compared to the control samples. All assays were performed in triplicate for each condition.

### Effects of rapamycin and curcumin on inflammation in the post-SE rat model for TLE

To study whether rapamycin and curcumin could be anti-inflammatory in early stages of epileptogenesis, we tested the effects of 1-week treatment with rapamycin or curcumin following electrically induced SE in rats.

#### Animals

Adult male Sprague Dawley rats (Harlan Netherlands, Horst, The Netherlands) weighing 250–350 g at the start of the experiment were used. Rats were housed individually in a controlled environment (21 ± 1 °C, 60% humidity, 12-h light/dark cycle with lights on 08:00 AM–8:00 PM), with water and food (standard laboratory chew) available ad libitum. All animal procedures were approved by the Animal Ethics Committee of the University of Amsterdam, according to Dutch law, and performed in accordance with the guidelines of the European Community Council Directives 2010/63/EU.

#### Status epilepticus induction

Rats were implanted with intracranial electrodes for stimulation and EEG recording using surgical procedures that are described in Additional file 1 and elsewhere [38]. After several weeks of recovery, the rats underwent tetanic stimulation of the angular bundle in the form of a succession of trains of 50-Hz pulses every 13 s. Each train lasted 10 s and consisted of biphasic pulses of 0.5 ms with a minimal intensity of 300 μA and a maximal intensity of 700 μA. Stimulation was stopped when the rats displayed sustained forelimb clonus and salivation for several minutes, which usually occurred within 1 h. If not, stimulation was continued but never lasted longer than 110 min. SE was defined electrographically by the occurrence of periodic epileptiform discharges (PEDs) of 1–2 Hz in the hippocampal EEG immediately after termination of stimulation. Behavior was observed during electrical stimulation and several hours thereafter.

#### Rapamycin and curcumin treatment

Stock solutions of 150 mg/ml rapamycin (LC Laboratories, Woburn, MA, USA) were prepared in 100% ethanol and stored at − 20 °C until use. Prior to use, rapamycin stock was diluted in a vehicle solution (5% Tween 80 + 5% polyethylene glycol 400) resulting in a 4% ethanol containing solution. Rats (*n* = 5) were injected intraperitoneally with 6 mg/kg rapamycin solution, starting 4 h after SE and once daily for 7 days thereafter. A vehicle post-SE group (*n* = 5) was injected with vehicle solution following the same paradigm. To allow for intracerebral ventricle (icv) injections for curcumin treatment, a stainless steel cannula was placed on the cortex of the rats during electrode implantation at 1.0 mm AP and 2.5 mm ML from Bregma and secured to the skull with dental cement. Curcumin (70%, Sigma-Aldrich, Zwijndrecht, The Netherlands) was stored at − 20 °C and freshly prepared (diluted in DMSO) on each treatment day. Rats (*n* = 7) were injected via the icv cannula with 2 μl of 2 mM curcumin solution, and a vehicle post-SE group (*n* = 4) was injected with a vehicle solution (DMSO, Sigma) using the same paradigm. Similar to the rapamycin treatment, curcumin injections started 4 h after SE and continued with daily injections for 7 days after SE. Based on the estimates of the cerebrospinal fluid (CSF) volume in the rat [39, 40], the end concentration of curcumin was estimated at a range of ~ 20–40 μM, which is comparable with in vitro levels reported previously [33] and in our vitro data in the supplementary results (Additional file 3: Figure S2). The end concentration of DMSO in the cerebrospinal fluid was ~ 1–2%. Control rats were included that were not stimulated or treated.

One week after SE, the rats were killed by decapitation under isoflurane anesthesia, and the hippocampal sections were dissected and stored in − 80 °C until use. This time point was chosen because it coincides with the activation of microglia and astrocytes and the upregulation of various pro-inflammatory markers, according to previous findings [41, 42].

### RNA isolation and quantitative real-time quantitative PCR

For RNA isolation, cell culture material was homogenized in Qiazol Lysis Reagent (Qiagen Benelux, Venlo, The Netherlands). Total RNA was isolated using the miRNeasy Mini kit (Qiagen Benelux, Venlo, The Netherlands) according to the manufacturer’s instructions. For rat hippocampal tissue sections, total RNA was isolated using the TRIzol® LS Reagent, following the manufacturer’s instructions (Invitrogen—Life Technologies, Breda, The Netherlands). Concentration and purity of RNA were determined at 260/280 nm using a NanoDrop 2000 spectrophotometer (Thermo Fisher Scientific, Wilmington, DE, USA). To evaluate mRNA expression, 200–500 ng (for cell culture-derived total RNA) or 2500 ng (for rat hippocampal total RNA) was reverse-transcribed into cDNA using oligo dT primers. Quantitative RT-PCRs were run on a Roche Lightcycler 480 thermocycler (Roche Applied Science, Basel, Switzerland) using the following primers: for human cell cultures: IL-6 (forward: ctcagccctgagaaaggaga; reverse: tttcagccatctttggaagg), COX-2 (forward: gaatggggtgatgagcagtt; reverse: gccactcaagtgttgcacat), EF1a (forward: atccacctttgggtcgcttt; reverse: ccgcaactgtctgtctcatatcac), and C1orf43 (forward: gatttccctgggtttccagt; reverse: attcgactctccagggttca). For rat hippocampal samples: IL-1β (forward: aaaaatgcctcgtgctgtct; reverse: tcgttgcttgtctctccttg), IL-6 (forward: gccagagtcattcagagcaa; reverse: cattggaagttggggtagga), TGF-β (forward: cctggaaagggctcaacac; reverse: cagttcttctctgtggagctga), Hmox-1 (forward: caaccccaccaagttcaaaca; reverse: aggcggtcttagcctcctctg), HMGB-1 (forward: gtaattttccgcgcttttgt; reverse: tcatccaggactcatgttcagt), cyclophilin A (CycA) (forward: cccaccgtgttcttcgacat; reverse: aaacagctcgaagcagacgc), and GAPDH (forward: atgactctacccacggcaag; reverse: tactcagcaccagcatcacc).

Quantification of data was performed using the computer program LinRegPCR in which linear regression on the Log (fluorescence) per cycle number data is applied to determine the amplification efficiency per sample [43]. The starting concentration of each specific product was divided by the geometric mean of the starting concentration of the reference genes (EF1a and C1orf43 for cell cultures and CycA or GAPDH for hippocampal sections), and this ratio was compared between groups. For the in vivo experiments, ratios were normalized to not-stimulated control group values. If treatment groups were not different from their corresponding vehicle group, the vehicle and treatment groups were pooled to make one post-SE group to compare to the corresponding not-stimulated control group.

### Western blot

Cells in culture or rat hippocampal tissue sections were homogenized in lysis buffer containing 10 mM Tris (pH 8.0), 150 mM NaCl, 10% glycerol, 1% NP-40, 0.4 mg/ml Na-orthovanadate, 5 mM EDTA (pH 8.0), 5 mM NaF, and protease inhibitors (cocktail tablets, Roche Diagnostics, Mannheim, Germany). Protein content was determined using the bicinchoninic acid method. For electrophoresis, an equal amount of proteins (10 or 15 μg/lane for cell cultures and rat hippocampal sections, respectively) were separated by sodium dodecyl sulfate-polyacrylamide gel electrophoresis (SDS-PAGE, 12% acrylamide). Separated proteins were transferred to nitrocellulose paper by electroblotting for 1 h and 30 min (BioRad, Transblot SD, Hercules, CA, USA). After blocking for 1 h in TBST (20 mM Tris, 150 mM NaCl, 0.1% Tween, pH 7.5)/5% non-fat dry milk, blots were incubated overnight at 4 °C with the primary antibodies (phosphorylated S6 (pS6) #5364, rabbit monoclonal, Cell Signaling Technology, Danvers, MA, USA, 1:1000; MAPK #9102, rabbit monoclonal, Cell Signaling Technology, 1:1500; phosphorylated MAPK (pMAPK) #4370, rabbit monoclonal, Cell Signaling Technology, 1:1500; β-tubulin, mouse monoclonal, Sigma, St. Louis, MO, USA; 1: 50,000). After several washes in TBST, the membranes were incubated in TBST/5% non-fat dry milk, containing the goat anti-rabbit or rabbit anti-mouse antibodies coupled to horseradish peroxidase (1:2500; Dako, Glostrup, Denmark) for 1 h. After washes in TBST, immunoreactivity was visualized using ECL Western blotting detection reagent (Thermo Fisher Scientific, Wilmington, DE, USA). For the culture experiments, for each condition, two wells were analyzed from a total of two donors.

### Data analysis

Data were analyzed using non-parametric testing (Kruskal-Wallis followed by Dunn’s tests for multiple comparisons, or Mann-Whitney *U* tests), using GraphPad™ Prism v.5 (GraphPad Software, Inc. La Jolla, CA, USA). *p* < 0.05 was assumed to indicate a significant difference.

## Results

### Effects of curcumin and rapamycin on inflammation and oxidative stress in vitro

**Fig. 1:**
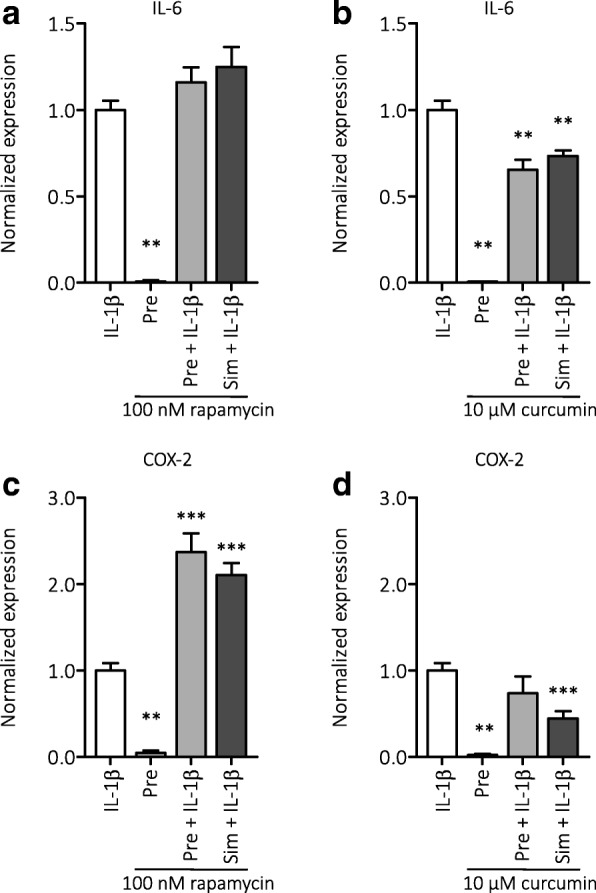
Anti-inflammatory effects of curcumin in cultured astrocytes that were challenged with IL-1β. **a** Rapamycin treatment did not affect the IL-1β-induced increase in IL-6 expression. **b** Curcumin, both before (pre-treatment) and simultaneous treatment with the IL-1β challenge, reduced the increase in IL-6 expression. **c** Rapamycin further increased the expression of COX-2 both after pre-treatment and simultaneous treatment. **d** Curcumin simultaneous treatment reduced the IL-1β-induced increase of COX-2 expression. Data are normalized to the IL-1β-stimulated condition (white bars) and shown as mean ± SEM. Pre = pre-treatment, sim = simultaneous treatment, **p < 0.01, ***p < 0.001 compared to the IL-1β-stimulated condition

Anti-inflammatory effects of rapamycin and curcumin were tested in vitro by measuring COX-2 and IL-6 mRNA expression in astrocyte cultures that were challenged with IL-1β stimulation (Fig. 1). IL-1β stimulation increased the expression of COX-2 and IL-6 in cultured astrocytes (*p* < 0.005 for both markers). The increase in IL-6 could be reduced by 10 μM curcumin, both with pre-treatment and simultaneous treatment (*p* < 0.01), whereas rapamycin did not affect the IL-1β-induced increase in IL-6 expression. The IL-1β-induced increase in COX-2 expression was reduced by simultaneous curcumin (10 μM) treatment (*p* < 0.005), but not by pre-treatment. Rapamycin (both pre- and simultaneous treatment with 100 nM) further increased the expression of COX-2, compared to IL-1β stimulation (*p* < 0.005).

**Fig. 2:**
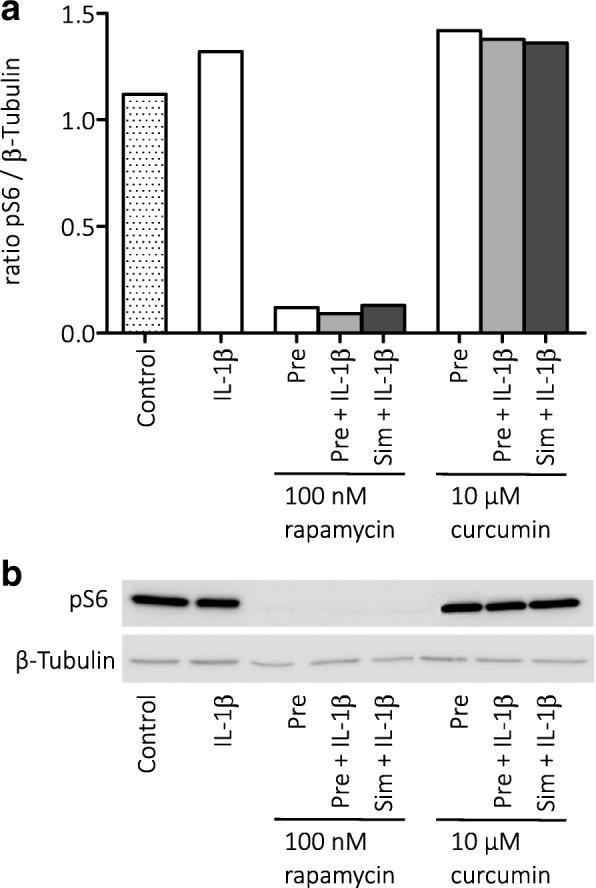
Effects of rapamycin and curcumin on the activation of mTOR in cultured astrocytes. **a** Rapamycin, but not curcumin, reduced the expression of pS6, indicating that rapamycin suppressed the activation of the mTOR pathway. **b** Corresponding Western blot image, pS6 (32 kDa) and β-tubulin (50 kDa) bands. Pre = pre-treatment, sim = simultaneous treatment

**Fig. 3:**
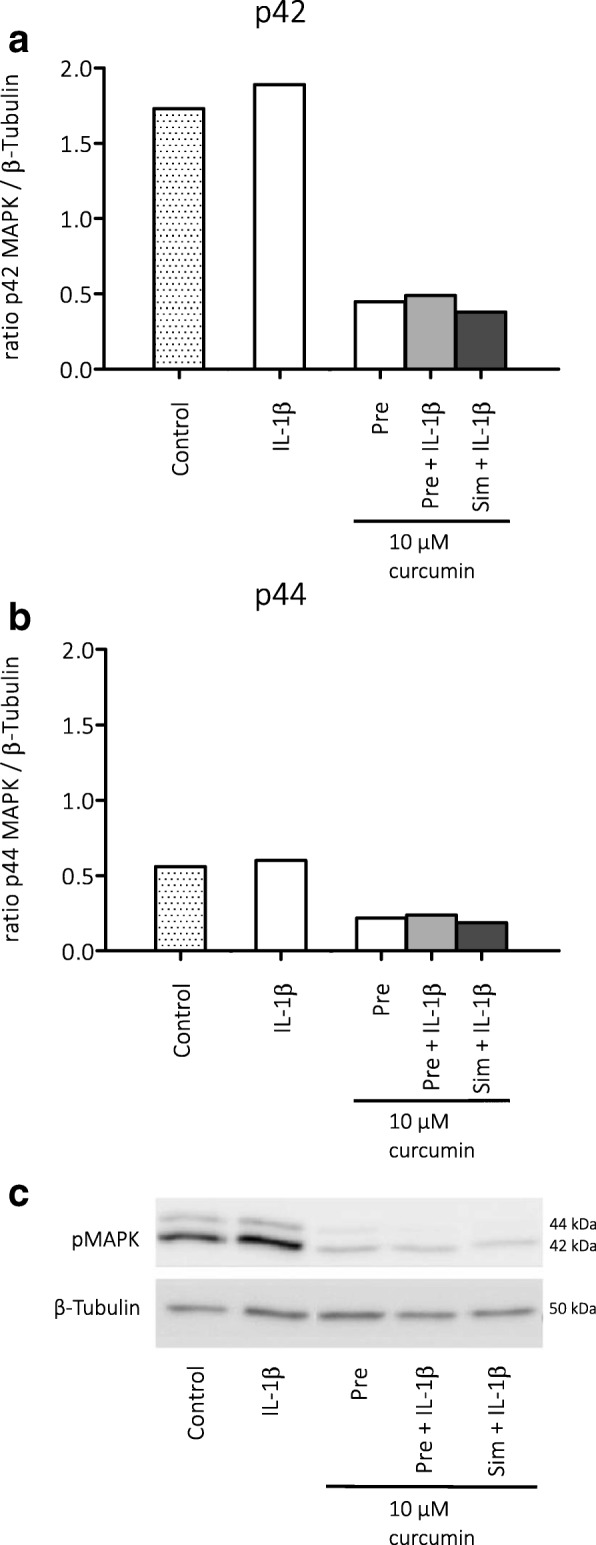
Effects of curcumin on activation of MAPK in cultured astrocytes. **a** Curcumin decreased the expression of p42 MAPK as well as **b** p44 MAPK, indicating that curcumin suppressed the activation of the MAPK/ERK pathway. **c** Corresponding Western blot image, pMAPK (42 and 44 kDa) and β-tubulin (50 kDa) bands. Pre = pre-treatment, sim = simultaneous treatment

To investigate the involvement of the mTOR pathway, the effect of rapamycin (100 nM) and curcumin (10 μM) on the downstream target of mTOR, phosphorylated S6 (pS6), was evaluated by Western blot (Fig. 2). Rapamycin, but not curcumin, suppressed pS6 levels in cultured astrocytes, in the presence of IL-1β, and without IL-1β stimulation. We next investigated whether curcumin could suppress the MAPK (ERK1/2) pathway, using Western blot for phospho-(p)MAPK (ERK1/2) and found that curcumin (10 μM) suppressed the levels of pMAPK in cultured astrocytes (Fig. 3).

**Fig. 4:**
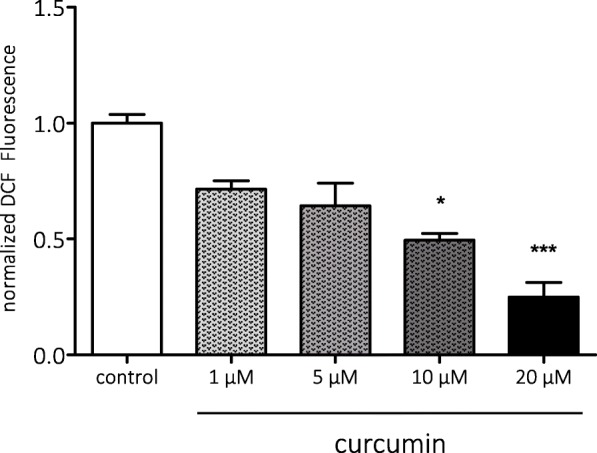
Anti-oxidant effects of curcumin in human SH-SY5Y cell cultures. The relative amount of DCF was dose-dependently reduced by curcumin, indicating that 10 and 20 μM curcumin reduced ROS in SH-SY5Y cell cultures. Data are normalized to control (0.05% DMSO—white bar) and shown as mean ± SEM from two separate experiments performed in triplicate (*p < 0.05 compared to control, ***p < 0.001 compared to control)

Next, we assessed the anti-oxidant effects of curcumin in vitro, by studying reactive oxygen species (ROS) in human SH-SY5Y cell cultures using a DCF assay. Curcumin (10 and 20 μM) reduced ROS levels after 30 min acute treatment compared to control (10 μM, *p* < 0.05; 20 μM, *p* < 0.001, Fig. 4).

### Effects of curcumin and rapamycin on inflammation and oxidative stress in post-SE rats

**Fig. 5:**
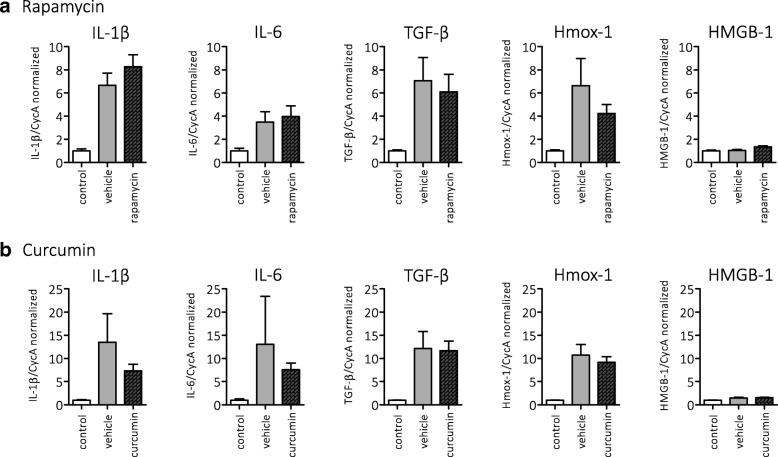
Inflammatory or oxidative stress markers were not reduced by rapamycin and curcumin in hippocampal tissue at 1 week after electrically induced SE. Expression of inflammatory markers interleukins IL-1β and IL-6, transforming growth factor (TGF)-β, and B oxidative stress marker Heme oxygenase (Hmox)-1 was increased at 1 week after SE in post-SE rats compared to not-stimulated controls but could not be reduced with **a** rapamycin or **b** curcumin treatment. High mobility group box 1 (HMGB-1) expression was not different between control and post-SE groups. Data are normalized to CycA and shown as mean ± SEM

We then set out to test the effects of rapamycin and curcumin in vivo, in the post-SE rat model for TLE. Rapamycin (6 mg/kg) and curcumin (~ 40 μM, see the “Methods” section) treatment was given for 1 week, starting 4 h after SE. Gene expression of inflammatory markers IL-1β, IL-6, TGF-β, Hmox-1, and HMGB-1 in the hippocampus was analyzed using quantitative RT-PCR 1 week after SE (Fig. 5). The expression of IL-1β, IL-6, and TGF-β was increased 1 week after SE compared to non-stimulated control rats. Expression of IL-1β, IL-6, and TGF-β was not different between treatment (rapamycin or curcumin) and their corresponding vehicle groups. Expression of HMGB-1 was not different between not-stimulated control rats and post-SE rats. Expression of Hmox-1 was increased 1 week after SE compared to non-stimulated control rats. Levels of Hmox-1 expression were not different between treatment (rapamycin or curcumin) groups and their corresponding vehicle groups.

### Effect of curcumin on pMAPK levels in post-SE rats

**Fig. 6:**
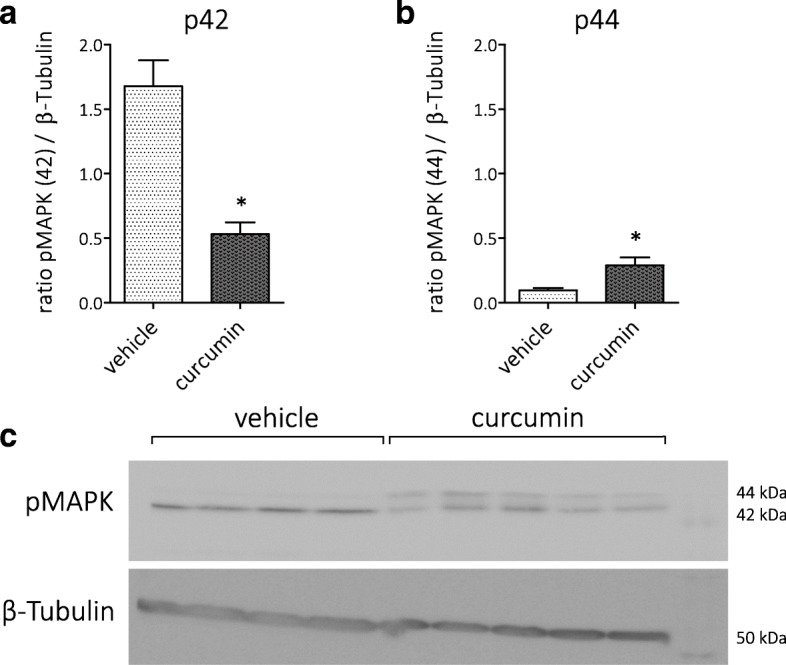
Effects of icv-applied curcumin on activation of the MAPK pathway in rat hippocampus 1 week after SE. **a**, **b** Curcumin reduced the expression of p42 MAPK and increased the expression of p44 MAPK, indicating that curcumin modified the activation of the MAPK/ERK pathway. **c** Corresponding Western blot image, pMAPK (42 and 44 kDa) and β-tubulin (50 kDa) bands (*p < 0.05 compared to vehicle)

To verify that icv-applied curcumin was able to inhibit the MAPK pathway in vivo, as was seen in vitro, hippocampal sections of post-SE rats were used for Western blot analysis. The 42-kDa band of pMAPK (p42) was suppressed in curcumin-treated versus vehicle-treated rats, whereas the 44-kDa band (p44 MAPK) was enhanced in the curcumin group (Fig. 6) indicating that curcumin reached the hippocampus in sufficient concentrations to influence the MAPK pathway.

## Discussion

We compared anti-inflammatory and anti-oxidant effects of curcumin and rapamycin to investigate whether curcumin has anti-epileptogenic potential that could make it an alternative for mTOR inhibitor rapamycin. We found anti-inflammatory and anti-oxidant effects of curcumin in vitro but not in vivo in the post-SE rat model.

When we assessed mTOR inhibition in vitro in the astrocyte cultures, we found that only rapamycin was able to reduce phosphorylated S6 levels, indicating that rapamycin, but not curcumin, inhibited the mTOR pathway. Instead, curcumin suppressed pMAPK (ERK1/2) in the astrocyte cultures. The activity of the MAPK pathway has been associated with different experimental models of epilepsy and seizures [44–50]. Increased MAPK activity could contribute to cell death [51, 52] and inflammation [53], and previous studies suggested that the neuroprotective effects of curcumin could be mediated by the MAPK pathway [26, 54, 55].

Anti-inflammatory effects differed between rapamycin and curcumin in the astrocyte cultures. Rapamycin did not suppress inflammatory cytokine IL-6 expression and even further increased COX-2 expression after the IL-1β challenge. This is in line with a previous study in rat astrocyte cultures that also showed increased COX-2 immunoreactivity [56] in microglia and increased IL-6 production after rapamycin treatment [57]. Since we used IL-1β as a trigger to mimic inflammation, we did not test rapamycin effects on the Il-1β mRNA level itself, but an effect by rapamycin on IL-1β and other inflammatory associated proteins (than IL6 and COX-2) cannot be excluded. Curcumin on the other hand suppressed IL-6 and COX-2, which is in line with the previously reported anti-inflammatory effects [23, 54, 55, 58, 59]. Curcumin only suppressed COX-2 when treatment was applied simultaneously with the IL-1β challenge. The findings that curcumin inhibits MAPK pathway and inflammatory cytokines in these experiments support the idea that curcumin could have anti-inflammatory effects mediated by the MAPK pathway in astrocytes, as suggested by previous studies [54, 55].

In SH-SY5Y cell cultures, we found anti-oxidant effects of curcumin, as shown by reduced ROS levels. Anti-oxidant effects of curcumin are likely mediated via induction of Heme oxygenase (Hmox-1), which can protect against damage by free radicals and programmed cell death [60]. Curcumin is a known inducer of Hmox-1 [27, 28, 61], which could contribute to its neuroprotective effects.

We then tested whether curcumin and rapamycin could interfere with inflammation during early epileptogenesis by measuring the expression of several inflammatory markers (after treatment during the first week after SE). Reduced inflammation would explain the seizure modifying effects of rapamycin observed later during epileptogenesis and would be in line with the anti-inflammatory actions of curcumin. At 1 week after electrically induced SE, there is an increase of activation of microglia and astrocytes, which is accompanied by the upregulation of various pro-inflammatory markers [41, 42]. Accordingly, we found that mRNA levels of inflammatory cytokines IL-1β, IL-6, and TGF-β were increased at this time point in post-SE rats compared to control rats. Upregulation of Hmox-1, indicative of oxidative stress, was also increased at 1 week after SE. We did not detect any effect of rapamycin or curcumin on inflammatory and oxidative stress markers at 1 week after SE. For rapamycin, this is in line with our findings in vitro. However, the lack of anti-inflammatory effects in vivo could also be explained by the recent findings that rapamycin can contribute to blood-brain barrier leakage at this early time point (but not in the chronic phase) after SE [16]. Blood-brain barrier leakage could initially contribute to the enhanced levels of inflammatory markers since it is known that peripheral immune cells and serum proteins that enter the central nervous system enhance inflammatory responses [62–64]. Alternatively, starting treatment 4 h after SE could have been too late for both curcumin and rapamycin to exert anti-inflammatory effects.

In another study using pentylenetetrazole (PTZ)-induced model for chronic epilepsy, curcumin did have anti-inflammatory and had anti-oxidant effects after oral administration [65, 66]. In the electrical post-SE rat model, however, we have not been able to detect effects of curcumin on the exhibition of spontaneous seizures when we administered curcumin orally [20]. Curcumin is known to have a low bioavailability due to rapid systemic degradation [31]. To circumvent these problems in the current study, we administered curcumin intracerebrally and found that curcumin did influence pMAPK at 1 week after SE but was not able to suppress brain inflammation. Possibly, SE induces too severe damage to be restored by curcumin treatment, or the dosing, location, and timing of the curcumin treatment were not sufficient to reverse the SE-induced inflammation and oxidative stress.

## Conclusions

Taken together, we found that 1-week post-SE treatment with rapamycin or curcumin did not alter SE-induced upregulation of markers of inflammation and oxidative stress, while in vitro, curcumin displayed anti-inflammatory and anti-oxidant effects. Since these in vitro results are promising, the anti-epileptogenic potential of curcumin deserves further investigation, possibly employing different TLE models and administration strategies to optimize bioavailability.

## Additional files

Additional file 1: Supplemental methods and results. (DOCX 46 kb)

Additional file 2: **Figure S1.** Effects of IL-1β, rapamycin, and curcumin on the viability of astrocyte cell cultures. (PDF 1690 kb)

Additional file 3: **Figure S2.** Cell viability of SH-SY5Y cells using MTT assay. (PDF 219 kb)

## Abbreviations

COX-2: Cyclooxygenase 2
DMSO: Dimethylsulfoxide
EEG: Electroencephalogram
ERK: Extracellular signal-regulated kinase
H2DCF-DA: 2′,7′-Dichlorofluorescein diacetate
HMGB-1: High mobility box group 1
Hmox-1: Heme oxygenase 1
icv: Intracerebroventricular
IL-6/IL-1β: Interleukin 6/IL-β
MAPK: Mitogen-activated protein kinase
mTOR: Mammalian target of rapamycin
ROS: Reactive oxygen species
SE: Status epilepticus
TGF-β: Transforming growth factor β
TLE: Temporal lobe epilepsy
