# Supplementary material for: Effects of rapamycin and curcumin on inflammation and oxidative stress in vitro and in vivo — in search of potential anti-epileptogenic strategies for temporal lobe epilepsy
Source: J Neuroinflammation. 2018 Jul 23;15:212. doi: 10.1186/s12974-018-1247-9 (PMC6056921; doi:10.1186/s12974-018-1247-9)
Supplement: Supplementary file 1 — Supplemental methods and results. (DOCX 46 kb) [file 12974_2018_1247_MOESM1_ESM.docx]

SUPPLEMENT

Effects of rapamycin and curcumin on inflammation and oxidative stress in vitro and in vivo – in search of potential anti-epileptogenic strategies for temporal lobe epilepsy

# Supplementary Methods

## Astrocyte cell cultures

Primary astrocyte-enriched cell cultures were made from human fetal brain tissue (cortex, 14-19 gestational weeks) obtained from medically induced abortions. A written informed consent for the use of the tissue for research purposes was given by all donors to the Bloemenhove clinic. Tissue was obtained in accordance with the Declaration of Helsinki and the Academic Medical Center (AMC) Research Code provided by the Medical Ethics Committee of the AMC. Cell isolation was performed as described elsewhere [1]. Briefly, after removal of blood vessels, tissue was mechanically minced into smaller fragments and enzymatically digested by incubating at 37˚C for 30 minutes with 2.5% trypsin (Sigma-Aldrich; St. Louis, MO, USA). Tissue was washed with incubation medium containing Dulbecco’s modified Eagle’s medium (DMEM)/HAM F10 (1:1) medium (Gibco, Life Technologies, Grand Island, NY, USA), supplemented with 1% penicillin/streptomycin and 10% fetal calf serum (FCS; Gibco, Life Technologies, Grand Island, NY, USA) and triturated by passing through a 70 µm mesh filter. Cell suspension was incubated at 37˚C, 5% CO_2_ for 48 hours to let glial cells adhere the culture flask before it was washed with phosphate-buffered saline (PBS; 0.1 M, pH 7.4) to remove excess of myelin and cell debris. Cultures were refreshed twice a week and reached confluence after 2-3 weeks. Secondary astrocyte cultures for experimental manipulation were established by trypsinizing confluent cultures and re-plating onto poly-L-lysine (PLL; 15 µg/ml, Sigma-Aldrich; St. Louis, MO, USA)-precoated 12 and 24-well plates (Costar, Cambridge, MA, USA; 10 × 104 cells/well in a 12-well plate for RNA isolation and quantitative real-time PCR; 5 × 104 cells/well in a 24-well plate for immunocytochemistry). Astrocytes were used for analyses at passage 2-4.

## Cell viability of astrocyte cell cultures – flow cytometry

The effects of different doses of rapamycin and curcumin on viability of the astrocyte cell cultures were analyzed. For flow cytometric analysis, cells were plated at a density of 10 × 104 cells/well in uncoated 12-well plates. Rapamycin (10, 100 or 500 nM) and curcumin (1, 10 or 100 µM) doses were diluted in DMSO, and the final concentration of DMSO was 0.05M for all treatments. Cells were treated with rapamycin or curcumin for 48h, or with 10 ng/ml IL-1β for 24h. Cells were detached from the culture plates, washed with PBS/1% bovine serum albumin (BSA) and stained with Fixable Viability Dye eFluor® 780 (1:2000, eBioscience, San Diego, CA, USA) for 30 minutes, after which the samples were washed again with PBS/1% BSA. Viability of cell cultures was determined by flow cytometric analysis using Fixable Viability Dye eFluor® 780 (eBioscience, San Diego, CA, USA). Flow cytometric analysis of stained cells was performed using a FACSCanto Flow Cytometer equipped with FACSDiva software (BD Biosciences) and data analysis was performed using FlowJo 7.6 (FlowJo LLC, Ashland, OR, USA).

## Oxidative stress assay in SH-SY5Y cells – DCF assay

Intracellular ROS levels were measured in SH-SY5Y cells using 2′,7′-dichlorofluorescein (DCF, Sigma-Aldrich, St Louis, MO, USA). Briefly, the cells were seeded in 96-well black fluorometric plates at a density of 10 × 10^3^ cells/well for 24 hours and then treated with different concentration of curcumin (1 µM-20 μM) as described in the preceding text. Successively, the cells were incubated with 10 µM 2′,7′-dichlorofluorescein diacetate (H2DCF-DA) in DMEM without phenol red for 30 minutes at 37 °C in the dark. After H2DCFH-DA staining, cells were washed twice with pre-warmed PBS and then re-suspended in DMEM without phenol red. The formation of DCF due to the ROS-driven oxidation of H2DCFH was measured using a microplate reader with excitation and emission wavelengths of 485 nm (bandwidth 5 nm) and 535 nm (bandwidth 5 nm) respectively.

## Cell viability of SH-SY5Y cells - MTT assay

The assay is based on the cleavage of the tetrazolium salt MTT in the presence of an electron-coupling reagent. Cell viability of SH-SY5Y cells was determined by the ability of mitochondria to convert 3-(4,5-dimethylthiazol-2-yl)-2,5-diphenyl tetrazolium bromide (MTT) in insoluble formazan. Briefly, cells were seeded in 96-well plates at a density of 10 × 10^3^ cells/well, incubated for 24 hours to let cells adhere to the plate and then treated with different concentrations of curcumin (1 µM - 20 μM) as described in the preceding text for 30 minutes. Subsequently, 15 µl of 5 mg/ml MTT reagent was added to each well and the plates were incubated for another 2 hours at 37 °C. The reaction mixture was then carefully discarded and 100 μl acid isopropanol (4mM HCl, 0.1% NP-40 in isopropanol) was added to each well to stop the conversion of MTT to insoluble formazan. Plates were kept in agitation at room temperature for about 15–20 minutes in the dark. The cell density was determined by measuring optical density (OD) at 570 nm wavelength using a microplate reader. Viable cells were quantified as a percentage and plotted relative to control. All assays were performed in triplicate for each condition.

*Implantation of electrodes for stimulation and intra-hippocampal EEG recording*

Rats were implanted with intracranial electrodes for stimulation and intra-hippocampal EEG recording, using the following surgical procedures: Rats were anesthetized with an intraperitoneal (i.p.) injection of ketamine (74 mg/kg; Alfasan, Woerden, The Netherlands) and xylazine (11 mg/kg; Bayer AG, Leverkusen, Germany) and placed in a stereotactic frame. In order to record hippocampal EEG, a pair of insulated stainless steel electrodes (70 μm wire diameter) was implanted into the left dentate gyrus under electrophysiological control as previously described [2]. The following coordinates were used: 3.9 mm anterior-posterior (AP), 1.7 mm mediolateral (ML) from Bregma. A pair of stimulation electrodes was implanted in the angular bundle at 7.2 mm AP and 4.5 mm ML from Bregma. Electrode pins were placed in a small six-pin connector and the assembly was attached to the skull with stainless steel screws and dental acrylic. After a minimum of two weeks recovery, rats were placed in their individual recording cages (40x40x80 cm) and connected to a recording and stimulation system (NeuroData Digital Stimulator, Cygnus Technology Inc., Delaware Water Gap, PA, USA) with a shielded multi-strand cable and electrical swivel (Air Precision, Le Plessis Robinson, France). Intra-hippocampal EEG was recorded continuously (24 hours/day) during baseline, SE induction and during 1 week following SE. EEG signals were amplified (10x) via a field effect transistor that connected the headset to an amplifier (20x; CyberAmp, Axon Instruments, Burlingame, CA, USA), band-pass filtered (1-60 Hz) and digitized by a computer. The signal was digitized by a computer and was sampled at 200 Hz per channel) by a seizure detection program (Harmonie; Stellate Systems, Montreal, QC, Canada).

# Supplementary Results

Viability of the human primary fetal astrocyte cell cultures was determined by flow cytometric analysis (supplementary figure 1). Following rapamycin and IL-1β treatments, viability of the cell cultures was not influenced, which was shown before [3]. Treatment with 1, 10 or 100 µM curcumin resulted in a cell viability of 95.7%, 91.6% and 52.8%, respectively (figure 1 d and e), suggesting that only 100 µM curcumin affected cell viability. In the following experiments 100 nM rapamycin and 10 µM curcumin were used. Cell viability was not affected by either of the curcumin doses (1, 5, 10 and 20 µM) in the SH-SY5Y cell cultures (supplementary figure 2).

# Supplementary Figure Legends

***Supplementary figure 1:* Effects of IL-1β, rapamycin (10, 100 and 500 nM) and curcumin (1, 10 and 100 µM) on viability of astrocyte cell cultures. A-D) Dot plots display the forward scatter versus eFluor fluorescence intensity of cells treated with vehicle (A), IL-1β (B) or the different rapamycin (C) or curcumin (D) concentrations. E) Bar graphs represent the percentage of viable cells (gated for low eFluor fluorescence) for all treatments.**

***Supplementary figure 2:* Viability of SH-SY5Y cells. The MTT colorimetric assay demonstrated no differences in percentage of viable cells between groups, indicating that cell viability was not influenced by curcumin. Data are normalized to control (0.05% DMSO – white bar) and shown as mean ± SEM from two separate experiments performed in triplicate.**

# References

1. van Scheppingen J, Iyer AM, Prabowo AS, Mühlebner A, Anink JJ, Scholl T, et al. Expression of microRNAs miR21, miR146a, and miR155 in tuberous sclerosis complex cortical tubers and their regulation in human astrocytes and SEGA-derived cell cultures. Glia. 2016;64:1066–82.

2. Gorter J a, van Vliet E a, Aronica E, Lopes da Silva FH. Long-lasting increased excitability differs in dentate gyrus vs. CA1 in freely moving chronic epileptic rats after electrically induced status epilepticus. Hippocampus. 2002;12:311–24.

3. van Scheppingen J, Broekaart DWM, Scholl T, Zuidberg MRJ, Anink JJ, Spliet WG, et al. Dysregulation of the (immuno)proteasome pathway in malformations of cortical development. J Neuroinflammation. 2016;13:202.
