# Supplementary figures and images for: Effects of rapamycin and curcumin on inflammation and oxidative stress in vitro and in vivo — in search of potential anti-epileptogenic strategies for temporal lobe epilepsy
Source: J Neuroinflammation. 2018 Jul 23;15:212. doi: 10.1186/s12974-018-1247-9 (PMC6056921; doi:10.1186/s12974-018-1247-9)

**A**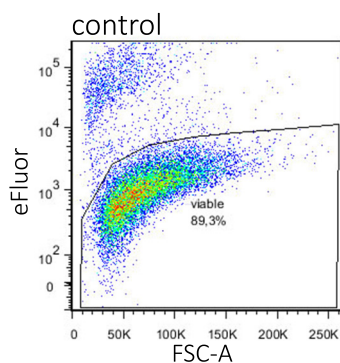**B**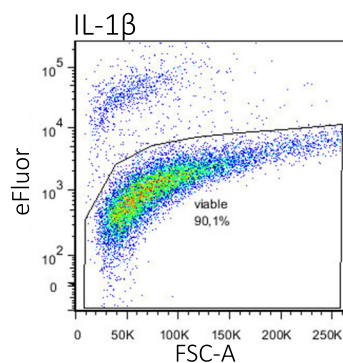**E**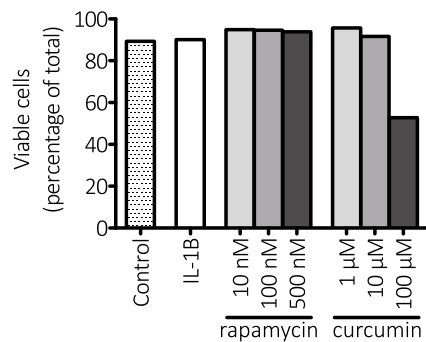**C**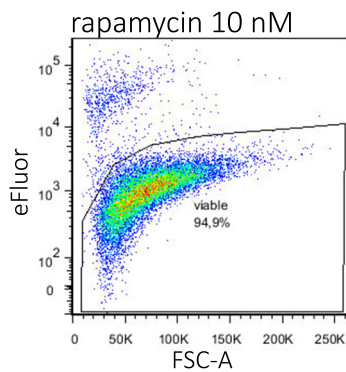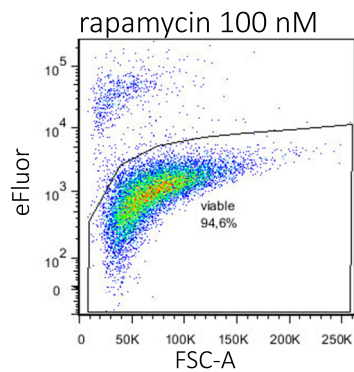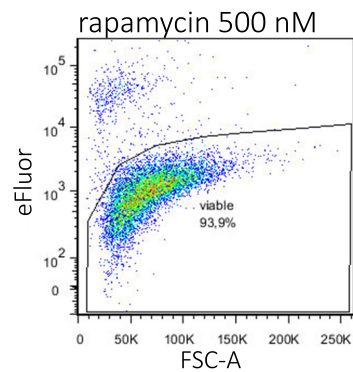**D**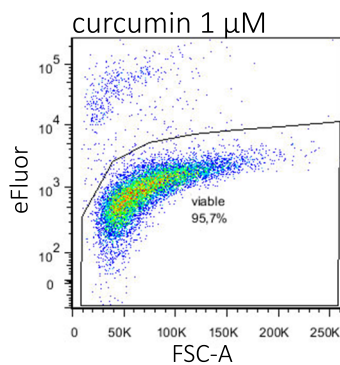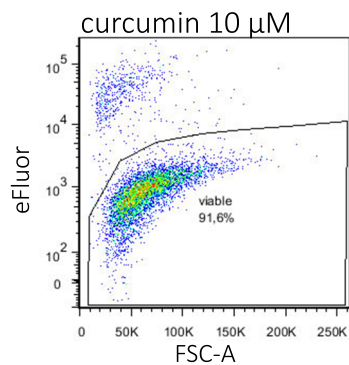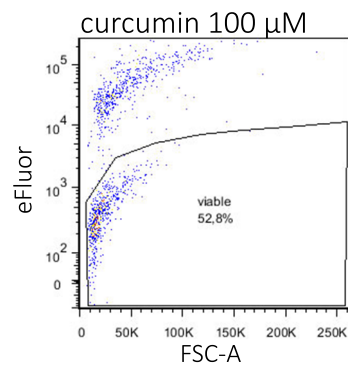

Supplement: Supplementary file 2 — Figure S1. Effects of IL-1β, rapamycin, and curcumin on the viability of astrocyte cell cultures. (PDF 1690 kb) [file 12974_2018_1247_MOESM2_ESM.pdf]

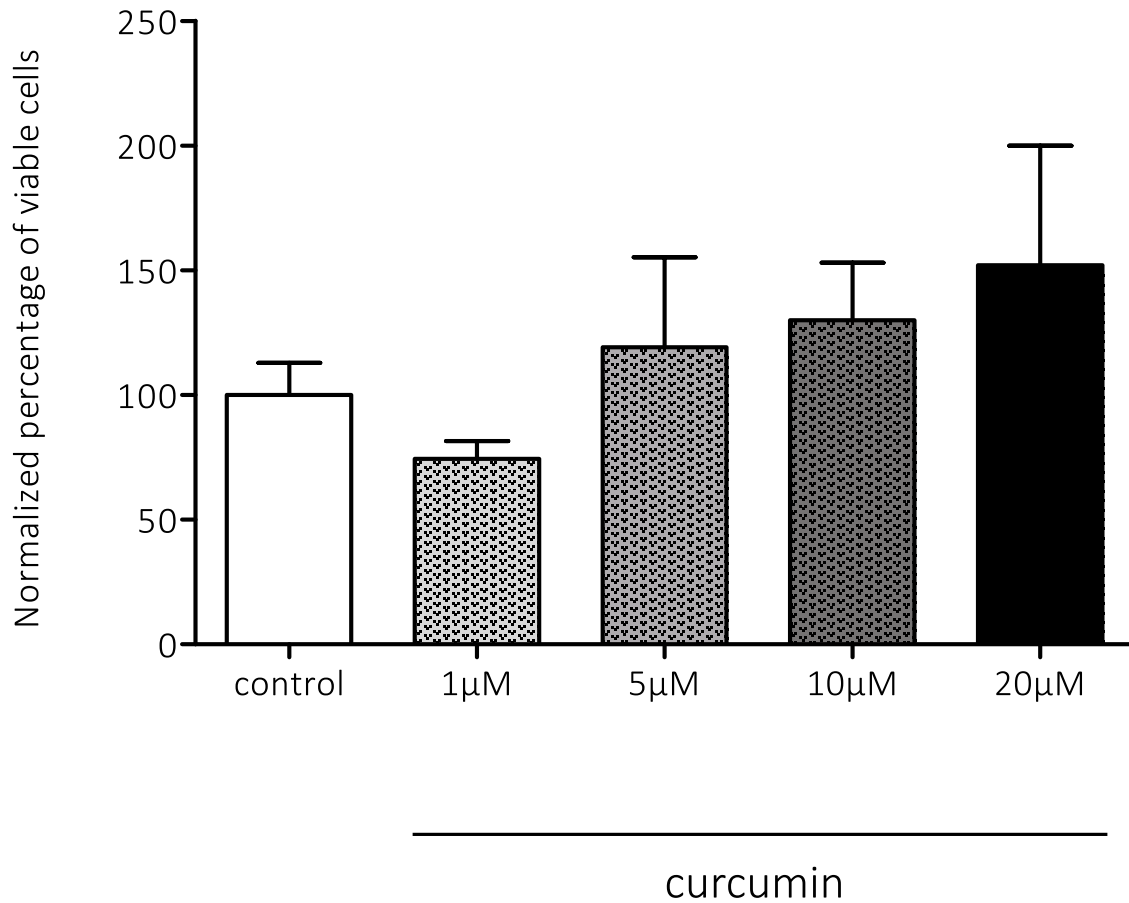

Supplement: Supplementary file 3 — Figure S2. Cell viability of SH-SY5Y cells using MTT assay. (PDF 219 kb) [file 12974_2018_1247_MOESM3_ESM.pdf]
